# Observations Respecting the Nature and Origin of the Common Species of Disorder of the Spine: With Critical Remarks on the Opinions of Former Writers on This Disease

**Published:** 1821-02

**Authors:** Edward Harrison

**Affiliations:** formerly President of the Royal Medical and Royal Physical Societies of Edinburgh, &c.


*Observations respecting the Nature and Origin of the common Species of Disorder of the Spine: with Critical Remarks on the Opinions of former Writers on this Disease.* By Edward Harrison, M.D. formerly President of the Royal Medical and Royal Physical Societies of Edinburgh, &c.

Of all the maladies by which mankind are assailed, none is more afflictive and distressing to relatives, or more deplorable and mortifying to the sufferers themselves, than spinal distortion. Persons so affected become objects of neglect or aversion; and, being rendered unfit to discharge with propriety the several duties of life, they remain burthens upon their friends. To a generous mind, nothing can be more painful and humiliating than to feel that in him the chief purposes of existence have been defeated, and that he is doomed to consume his days in wearisome inactivity, or in unproductive occupations.

That such must be the necessary consequences of this disease will be evident, if we take a survey of the important offices of the spine, and of its influence over the complicated functions of the thoracic, abdominal, and pelvic viscera. Curvatures in the cervical bones produce numbness and paralysis of the arms. Injuries received in any part of the spine induce loss of sensation, and of power of voluntary motion in the lower limbs.

Different internal organs are made to participate, according as the malady is seated higher or lower in the back. Pressure from dislocation of the cervical vertebræ impedes deglutition and respiration, by reducing the diameter of the œsophagus and trachea, as well as by disturbing the actions of their muscles and nerves. Diminished capacity of the chest, the unavoidable consequence of its deformity, gives rise, by displacing or squeezing the enclosed parts together, and preventing the convenient exercise of their functions, to palpitations of the heart, to dyspnœa, cough, and various distressing perceptions, which are followed, in process of time, by organic derangements of the heart and lungs, by consumption, asthma, hydrothorax, and other chronic disorders. When the luxated dorsal vertebræ compress the spinal cord, there is generally a constrictive uneasiness about the stomach, and a depraved secretion in some of the abdominal organs. Bony distortions of the thorax mechanically force the liver, the stomach, and the contiguous viscera, out of their natural places: hence arise painful sensations in them, and irregularity in their functions. The impediments thus created are followed by organic mischief in the parts, which terminates at length in the most distressing complaints. A displacement of the lumbar and sacral bones leads, in the same way, to very obstinate affections of the bladder, rectum, uterus, and external genital members.

Till the true nature of the disorder has been discovered in these cases, the most discordant opinions are advanced by the faculty, and the patients are tortured by the most opposite practices. A malady which is attended with the most disgusting appearances, and produces such a variety of obstinate symptoms, cannot be too soon or too carefully resisted. A vigorous opposition to the very *dawn* of the complaint is of greater consequence, because no effectual means have hitherto been attempted to remove the deformity, arrest its progress, or avert the maladies which proceed from it. In order to show the truth of these remarks, and prepare the reader for a more successful method of cure, I propose to give a short account of the principal writers who have delivered their sentiments upon this disorder, their respective opinions, and the different practices which they have employed to subdue it.

Society is under great obligations to the late Mr. Pott, for calling the attention of his brethren to an investigation of the causes and treatment of one of the most obstinate and distressing complaints to which the human frame is exposed. This subject, though much agitated in the early ages of mankind,* had been unaccountably neglected in modern times. Mr. Pott

* See Galen, Avicenna, &c.

brought it again before the public, in his valuable remarks on Palsy of the Lower Limbs.* So much deference was paid to his appeal, that he had the satisfaction immediately to succeed in rescuing several miserable victims out of the hands of unfeeling and rapacious quacks, who had till then ingrossed to themselves the whole care and management of these unfortunate sufferers. The eminent station of this accomplished surgeon enabled him to treat many patients, and enrich his Remarks with several dissections of persons who had sunk under the complaint.

"The true cause of the disease is," he observes, "a morbid state of the spine, and of some of the parts connected with it; which distempered state of parts will, upon careful inquiry, be always found to have preceded the deformity some length of time." "A morbid state of parts previous to deformity, caries, or curve, must be allowed: every complaint of the living, and every appearance of the dead, prove it beyond contradiction or doubt. All the general complaints of persons afflicted with this disorder will always, upon careful inquiry, be found to have preceded any degree of deformity, to have increased as the curve became apparent, and to have decreased as the means used for relief took place. The pain and tightness about the stomach, the indigestion, the want of appetite, the disturbed sleep, &c. gradually disappear, and the marks of returning health become observable, before the limbs recover the smallest degree of their power of moving."

"That this curvature, which is always from within outward, is caused by the erosion or destruction of part of the body, or bodies, of one or more of the vertebræ; by which means, that immediately above the distemper, and that immediately below it, are brought nearer to each other than they should be; the body of the patient bends forward, the spine is curved from within outward, and the tuberosity appears behind, occasioned by the protrusion of the spinal processes of the distempered vertebræ."

"That, without this erosive destruction of the bodies of the vertebræ, there can be no curvature of the kind which I am speaking of,—or, in other words, that erosion is the *sine qua non* of this disease; that, although there can be no true curve without caries, yet there is, and that not unfrequently, caries without curve."†

These passages might be confirmed by reference to several others. Having extracted them from his last essay on Spinal Maladies, it is reasonable to conclude that they contain a faith-

* Chirurgical Works of Percival Pott, Esq. vol. iii.

† Pott on the Palsy, &c. vol. iii. pp. 427, 457, 486, 493.

ful record of his latest sentiments on the subject. Had any subsequent change of opinion taken place, it would doubtless have been recorded in the "Notes and Observations" of his respectable editor and relative, Sir James Earle.

It appears from these extracts, and from the spirit of Mr. Pott's writings, that, in his estimation, caries formed a necessary and essential part of this formidable malady. We shall take another opportunity to prove that paraplegia can appear in its most complete form, independently of caries; and it is equally certain that caries of the vertebræ* is frequently unaccompanied with paraplegia.

We are instructed in the foregoing passages, that spinal complaints affect three different textures,—the bones, ligaments, and cartilages. The last are brought to participate during the progress, and cannot therefore be allowed any share in the original formation. Though Mr. Pott always found disease in the "ligaments,"† and "sometimes in them without any apparent affection of the bones,"‡ he unaccountably overlooked them, and limited his curative indications to the morbid state of the bones. In so doing, he reasoned upon a narrow basis. Admitting his own statement in its full extent, I think we are still bound to pay some regard to the ligaments. It however appears, from later dissections, that the vertebral bones are not always enlarged, or otherwise disordered, even when they are accompanied with a greater or less degree of deformity and crookedness of the spine.§ Many skeletons, showing lateral, posterior, and anterior curvatures, may be seen in the anatomical museums; several of them so exceedingly mis-shapen, as to leave no doubt of their having occasioned death, by interfering with the functions of the spinal marrow, the thoracic and abdominal organs. In some, the vertebral bones and cartilages show no other signs of disease than such as arise from unequal pressure: they are hard, firm, sound, and, where free from pressure, are of the natural size. In order to form a curvature, the bones and cartilages must necessarily be fuller on the outside and compressed on the inside. The bones are even unnaturally separated in some parts of the external bow, yet nothing like ulceration had taken place.

According to this view of the subject, we must direct our attention to some other tissue to discover the true cause of spinal complaints; and I am of opinion that we shall find it in the connecting ligaments, "which seem to have lost part of their power of holding the bones together."|| These get relaxed,

* Lieutaud, Morgagni, *Lond. Med. Observat.*, De Hæn, Stork, Bacchinus, Lecat. Aubertus, Blanchard, Hildanus.

† Pott, vol. iii. p. 427, 428, 438. ‡ Vol. iii. p. 482.

§ Vol. iii. p. 483. || P. 438.

and suffer a single vertebra to become slightly displaced. The column now losing its natural firmness, other bones begin to press unduly upon the surrounding ligaments; they, in turn, get relaxed and elongated, by which the dislocation is increased and the distortion permanently established. The direction becomes lateral, anterior, or posterior, according to circumstances; but the malady has in every instance the same origin, and requires the same mode of cure.

It appears to me that, when the spinal trunk gets too infirm to maintain the weight of the head and shoulders, the distortion inclines to the lateral form, being encouraged to take that direction from the resistance given by the superior spinal ligament. The anterior or internal curve, as far as my experience extends, has always been confined to the lower cervical and upper dorsal, or to the lumbar, vertebræ. The spine is depressed in both instances, appearing to have sunk under and been crushed downwards by an oppressive and overwhelming burden. When it arises, in a weakly constitution, from the exertion of pulling, lifting, or carrying, the protrusion, from the force employed, is nearly backward; and thus is laid the foundation of the posterior or outward curve. This is the simple and real origin of our spinal deformities, except in some rare instances, where they begin in the vertebral or cartilaginous structure. On this principle, we can easily understand why people so often recover from spinal affections, and carry about with them the visible effects for many years. If the primary mischief begun in bone, as Mr. Pott teaches, or in cartilage, according to others, I think the affection would be very seldom cured, either by the unaided efforts of nature or the best assistance of the healing art. "No degree of benefit or relief, nor any the smallest tendency towards a cure, is to be expected until the caries be stopped and the rotten bones have begun to incarn: the larger the quantity of bones concerned, and the greater degree of waste and havoc committed by the caries, the greater must be the length of time required for the correction of it, and for restoring to a sound state so large a quantity of distempered parts."* When any thing like this degree of caries and rottenness occurs in the living body, the matter generated can never be absorbed. In defiance of the best efforts of the constitution, it will most certainly burst forth and lay the foundation of very tedious, if not fatal, drains from the back, groin, thighs, or hips.

The practice of Mr. Pott arose, by an obvious induction, from his own premises. His object was to excite inflammatory action by caustic issues, and thereby induce anchylosis, or

* Pott, vol. iii. p. 474.

union among the morbid bones. That such treatment is useful at an advanced period, after caries is actually formed, may be agreeable to sound practice, though it has never obtained the universal approbation of medical men. Professor RUST* remarks, that numerous observations and long experience have proved to him that issues rarely produce the desired effect, and that they even frequently accelerate the progress of the disease in a late stage. At an early period, while the disorder is confined to ligament alone, the practice of Mr. Pott is highly objectionable, because it prevents the application of other modes better calculated to restore the sufferer to his natural figure and former health. It cannot be denied, nor do I wish to insinuate, that patients have not got well upon this plan. Caustics, by stimulating, encourage the muscles and ligaments to act more energetically, by which they sooner regain their lost tone and vigour. The curative process is further expedited by the rest to which invalids must to a certain degree submit, while smarting under the pain of caustics. In many cases, Mr. Pott found it necessary to do more than employ caustic issues: he actually confined his patients to bed or to an horizontal situation, during the greatest part of the cure, as they could not bear to remain in an upright position.† In all these recoveries, the subjects of them remain through life in puny health, because, the bones continuing displaced, and some of the viscera being necessarily subjected to injurious pressure, the important functions of the spinal cord are imperfectly discharged, owing to the difficulties it meets with from the altered form and direction of the medullary canal.

"The deformity remaining after recovery is subject to great uncertainty and considerable variety, as it depends on the degree of caries and the number of bones affected: in general, it may be said, that where one vertebra only is affected, and the patient young, the curve will in length of time almost totally disappear; but, where two or three are affected, this cannot be expected. The thing aimed at is the consolidation and union of the bones, which had been carious and are now become sound. This is the *sine qua non* of the cure, and this must, in such cases, render the curvature, and consequently the deformity, permanent. The issues will restore the use of the limbs, but not the lost figure of the spine."

"That, when two or more vertebræ are affected, forming a large curve, however perfect the success may be with regard to the restoration of health and limbs, yet the curvature will, and must, remain, in consequence of the union of the bones with each other.‡

* Professor Rust's *Arthrocacology*.

† *Observations on the Cure of the Curved Spine, &c.* by James Earle, Esq. p. 28.

‡ Pott, vol. iii. p. 478, 491.

It is clearly apparent, from the foregoing quotations, and the candid admissions of Mr. Pott, that he never attempted to restore the back to its natural figure. Such an happy result never came under contemplation; nor could it, consistently with his ideas, form part of the curative plan. Believing, as he did, that the mischief always begun in the bodies of the vertebræ, his efforts were confined to prevent ulceration, to cure it when present, and to join the bones permanently together in their curved form by an immovable union.

Had Mr. Pott attended carefully to the incipient stage, it is more than probable he would have found that the affection generally begins in the ligaments, and passes from them to the bones and cartilages. It is of the greatest importance to keep this distinction constantly in view, because the knowledge of it leads to a marked difference in practice. At this period, the complaint, according to my experience, is more certainly curable than many others; and, what is most encouraging, *the spinal column is made wholly to recover its natural form and powers.* Instead, therefore, of sustaining the miseries of an infirm constitution for the rest of life, and carrying deformity into all companies, the individual returns into society in as perfect health and shape as if he had never been afflicted.* That such has been the pleasing result of the new method, is abundantly proved by experience, and can be confirmed by many living witnesses. Had the disorder, in any of these examples, been attended with caries, very different must have been the result. The complaint, instead of submitting to the means employed, would either have gradually increased, or, had a cure been at last obtained, there would, for obvious reasons, have been no reduction in the tuberosity. The inevitable consequences must have been a mis-shapen form, and its attendant evils.

A cure conducted agreeably to the foregoing principles leaves the patient in good health, and in the enjoyment of his natural shape. Where it has been produced by issues, how different is the result! In such instances, the curvature, and consequently the deformity, remain permanent.

"These observations,"† says Sir James Earle, "and others of the same tendency, added to the many melancholy instances which I have seen after a cure has been effected by issues alone, had long obtruded unpleasantly on my recollection. Prolonging life merely to lengthen out a miserable existence, and enable a wretched being to crawl a little longer on the earth, appears very unsatisfactory, and stopping short, if the idea be

* This subject will be prosecuted in successive Essays, till the author shall have fully explained his ideas of the disorder and its treatment.

† *Observations on the Cure of the Curved Spine, &c.*

indulged, that, by any assistance from art, more may be done. I have often thought it would be a most happy circumstance could we go a step further, and *cure the deformity so well as the disease;* for, besides the disgusting appearance of the crookedness which remained, want of health, debility and inactivity, usually accompanied it: and another very material consequence resulted from it,—namely, that the largeness of the remaining curvature rendered the spine mechanically weak; which, probably, being added to a tendency to the same softness of bones as was the foundation of the original malady, was the efficient cause of the disease being liable to return. This was a very strong and additional reason why the improvement of the form of the spine should be equally the object of our attention with the cure of the disease."

It does not excite surprise that Mr. Pott, chiefly conversant with the worst and most obstinate cases, was led to believe that the disorder always begun in the bones. These were, as he thought, enlarged, and pressed upon the cord. Having been induced, for reasons which he has not fully explained, to abandon this opinion, Mr. Pott did not afterwards attempt to account for loss of motion in the limbs. He contented himself with asserting that it bore no resemblance to the true paralysis of the same parts. Sir J. Earle, in his anxiety to supply the defect, ventured to go no farther than to revive the discarded opinion of his preceptor. I mention this circumstance as a proof that little progress had been made in the pathology or treatment: the great authority of the master seemed for a time to over-awe and bear down opposition.

One of our latest writers imputes the disorder to pressure from an inflammatory increase of bulk in the fibro-ligamentous substances interposed between the vertebræ. He was induced to give up the opinion of Mr. Pott, because bony enlargements of the spine are seldom found in the dead body.* We have little difficulty in joining our testimony to that of Mr. Copeland, in cases of external violence. It is, however, uncertain whether in these instances the inflammation commences in bone, cartilage, or membrane. The species to which I wish to draw attention, makes its attack so insidiously as not to be discovered till a long time after its invasion. It exhibits none of the local characters of inflammation, nor is it accompanied with pyrexia. This, which is the common variety, seizes upon relaxed fibres, or persons accidentally debilitated. I am enabled, from the treatment of many such cases, to assert that they are wholly unconnected with inflammatory diathesis, and very seldom originate in a morbid condition of the vertebræ.

* Copeland on Diseased Spine. Gortz *de Morbis Ligamentorum.*

In order to understand the true origin and injurious effects of these disorders, we must premise a cursory survey of the operations which the spinal column and its contents are destined to perform in the living animal. The nine or ten pairs of nerves which issue immediately from the brain, seem to be chiefly occupied with conveying perceptions to the sensorium commune. The thirty-nine or forty pairs which arise out of the spinal marrow, communicate the power of feeling and voluntary motion to the limbs and muscles of the trunk and head. They, in conjunction with the ganglions and great sympathetic nerve, supply the sanguiferous system, the thoracic and abdominal viscera, with the principal share of their nervous influence. By experiments performed upon living animals, we learn that these several organs continue to execute their appropriate functions long enough after all connection with the brain has been destroyed by decapitation, to induce us to believe that they derive their predominant energy from a different source. This seems to be the spinal marrow; for, when it is removed, the abdominal and thoracic organs soon cease to act. We are therefore led to conclude, that, although the animal functions are performed by the brainular nerves, those of organic life, or such as are only intended to support the machine, are executed by the spinal nerves. In this view of the subject, an healthy disposition of the spine is indispensable to the well-being of the individual. That the inference rests upon experience we shall be able to prove, by taking a review of the principal symptoms which occur in this disorder. When it affects the superior cervical bones, deglutition is impaired, from pressure made upon the cervical nerves in their course to the pharynx and œsophagus. For the same reason, a dry teazing cough and difficult breathing are occasioned by pressure upon the nerves in their way to the larynx and trachea.

Projections in the cervical bones affect the arms with numbness, debility, spasmodic twitchings, and paralysis. They also produce uneasiness in respiration, with palpitations from slight causes. In the dorsal vertebræ, they induce a girding sensation over the stomach, as if it were tied with cords. There is also indigestion, and oftentimes vitiated appetite. The secretion of bile becomes diminished, the countenance looks sallow, and the patient labours under many symptoms of jaundice. The belly is obstinately constipated, and the rectum refuses its office. The feces are often slimy, whitish, or clay-coloured. He gets feverish, is restless, emaciated, and affected with many symptoms, which resemble consumption. The kidneys secrete little urine, and the bladder loses its expulsive faculty. In whatever part of the back the curvature is situated, the lower limbs are apt to be affected. Slight pressure upon the spinal cord produces debility and fatigue in walking. The legs cross each other; the patient is liable to stumble, and cannot go straight to any point. Greater pressure occasions muscular spasms, numbness, restlessness, and clammy sweats. A still greater pressure produces inability of motion, and complete paralysis of the limbs.

The numerous, obstinate, and complicated maladies, enumerated above, are truly appalling. They exhibit spinal complaints in a new light, and claim for them an attention which they have not hitherto received. Whether we consider them as giving an unseemly appearance and laying the foundation of delicate health, or regard them as the fruitful parent of many other formidable disorders, the subject is worthy of the most rigorous investigation. Happy will it be for our contemporaries and successors, if a complaint almost universally prevalent in the middle and higher classes can be subdued so as to leave behind it no vestige of its former existence. In order to accomplish the object, we must endeavour to make out the cause by tracing the different and apparently-unconnected symptoms to their true source. Perplexing as the inquiry may seem at first sight, we are inclined to believe that it admits of explanation upon anatomical principles. In the natural form of the back, the nerves meet with no impediment in their passage from the cord through their proper foramina in the several vertebræ. Distortion of the spine produces an alteration in the canal under it, and the vertebral holes are necessarily forced into unnatural directions. In consequence of these changes, the tender substance of the cord gets pressed against the hard sides of its sheath. The nervous filaments which issue from it, having to travel over a longer course, become unduly stretched, and encounter angles and projections in their way through the bony holes of the vertebræ, by which their energies are impaired, interrupted, and morbidly affected. We have already shown that the spinal nerves have been traced to all the abdominal and thoracic organs, to the sanguiferous system, to the upper and lower extremities ; also to the trunk of the body, the neck, and outside of the head. When we contemplate this distribution upon anatomical and pathological principles, we cease to be surprised that luxations of the vertebræ should produce such an endless train and succession of perplexing symptoms. The extensive range of the spinal nerves, and momentous offices they are destined to perform in the animal fabric, afford an easy solution of our difficulties. Not that all the symptoms and sufferings enumerated were ever encountered by any individual.

It has been already declared that the symptoms vary according to the situation of the curve. Organs supplied with nerves from a higher part of the spine than the seat of disease, are seldom affected. In slight cases of pressure, even the thoracic and abdominal members, which derive their nervous influence from below the distortion, are but little disturbed. The symptoms, in all these instances, are chiefly confined to the lower extremities and the viscera which receive their nerves immediately from the spinal curvature. In order more clearly to explain this part of our subject, we must premise, that the neck consists of seven flexible vertebræ; the back contains twelve, and the loins five. From each of these vertebræ issues a pair or bundle of nerves; one portion of which diverges to the right, another towards the left side, and the remainder run into the body. From the os sacrum arise five more pairs of nerves. It follows from this calculation, that thirty-nine pairs, or seventy-eight nervous cords, each including many distinct filaments, originate in the spinal marrow. Having passed through as many proper holes in the vertebræ, they separate into minute fibres, and are distributed over the greater part of the body, to regulate and control the most useful and necessary functions of animate beings. When any of the vertebræ become displaced or too prominent, the patient experiences inconvenience from a local derangement in the nerves of the part. He, in consequence, is tormented with a train of nervous symptoms, which are as obscure in their origin as they are stubborn in their nature: they have therefore been justly denominated the *opprobia medicorum.* A sedulous examination into morbid anatomy having enabled us to disclose the latent sources of other ailments formerly concealed in impenetrable darkness, we may look forward with confidence to a similar result with regard to several nervous complaints, by directing inquiry to the spinal column and its delicate contents.

According to this view of the subject, the obvious indication for the cure of spinal affections consists in restoring the displaced bones to their natural situations, that the spinal cord and its nerves, relieved from injurious pressure and disturbance, may be re-instated in their former abilities. In all the cases hitherto treated agreeably to these principles, the success has been complete. The affected organs to which they run, being no longer under the influence of diseased nerves, gradually recover their healthy state and proper functions.

Mr. Baynton, having been frequently disappointed in the cure of these complaints by treating them according to the ordinary modes, was led to devise another course. He has favoured us with his practice in twelve spinal cases: of these, one died; eleven recovered and regained the use of their limbs. The process took up from seven to fifteen months, and the projection was reduced in every instance; in some it is asserted to have been removed. He imputes the want of success, where his treatment failed, to previous anchylosis of the displaced bones. These cases, partly drawn up by himself, and partly by unprofessional friends, are not sufficiently detailed and circumstantial to enable us to form a clear and satisfactory opinion either in regard to the process adopted, its success in reducing the enlargement of the back, or the degree of constitutional health to which the patients were afterwards restored. In all these particulars the treatise is very defective and unsatisfactory. We are, however, under great obligations to the candid author for making us acquainted with a plan which promises to be extremely advantageous in the management of these and other obstinate complaints. It consists in placing the patient horizontally upon a firm and unyielding mattress, where he is to remain constantly recumbent during the whole process. He is not accommodated with a pillow to support the head; nor is he to be moved in the least, for the most necessary occasions. All fears of the health suffering under this mode have been happily removed, by the successful issue of the different trials. In every case where it has been properly conducted, the patient soon became easy, cheerful, and regained his rest. Appetite and digestion improve under the confinement. In general, there is increase of flesh, and a marked improvement of countenance.

The intention of the plan is to afford the "softened bones of the vertebræ" an opportunity for recovery, and make themselves able to support the weight of the parts above. So long as the column remains perpendicular, it is capable of bearing considerably more weight than after it has become oblique. In order to restore the strength of the spine, the person is to be laid flat, to give the "softened bones of the vertebræ" a better opportunity to recover themselves. Until the enfeebled spine is relieved from its natural load it cannot get tone, because the weight of the head and shoulders prevents the benefit to be expected from any mode of treatment. A system of resting will accomplish cures after the failure of drains and machinery. It may be observed, that Mr. Pott, though he takes little or no notice of the matter, thought it necessary to confine his patients in bed, or the horizontal posture, during the greatest part of the cure, as they could not bear to remain in an upright disposition.* The length of time required to produce the cure, varies, as we have already stated, from seven to fifteen months. At the end of the limited period, the patients were allowed to rise and take exercise, according to their ability. We have not been informed by the author of any relapses, but such occurrences are

* See Sir James Earle's *Observations on the Cure of the Curved Spine.*

asserted to have happened from other quarters. They were probably owing to the want or neglect of proper directions in regard to the care and precautions which should be observed for some time after the reclining method has been discontinued. No means were used to remove the tenderness and pain of the back. These were soon subdued by rest alone. In no instance were external applications wanted. Internally, the liquor calcis muriatæ was administered, to increase and consolidate the ossific process. Besides this remedy, bark, and medicines to obviate costiveness, were also employed. It is difficult to say how long the resting should be continued; nor can we lay down any rules with confidence till we have had greater experience. In the slightest cases, the recumbency must be continued five or six weeks after the removal of all tenderness. Where disorganization of the bodies of the vertebræ has taken place, the rest must be prolonged two or three months after every inconvenient symptom has disappeared.

Such is the plan recommended by Mr. Baynton. It consists, as we have already observed, in placing the patient horizontally upon a mattress, without bolster or other elevation, and continuing the same position till the spine shall have recovered firmness enough to support the head, shoulders, and chest. This is the whole object contemplated, and such are the means employed to attain it. When the process is finished, no measures of precaution are advised to prevent relapses. The patients arise from their couch, and thus ends the treatment.

Mr. Earle, laudably solicitious to maintain the high character of his deceased ancestor, has for that purpose voluntarily entered the field of controversy with the Editors of the Medical and Surgical Journal.* In the review of Mr. Baynton's essay,† they had taken occasion to extol his "humane" method, compared with the severity of Mr. Pott's treatment of spinal complaints. In order to bring the matter fairly before an impartial public, Mr. Earle was led to analyze Mr. Baynton's twelve cases, published in favour of the resting process, and to accompany each of them with his own remarks. He reports that only three of them are of the description alluded to by Mr. Pott; and in them the previous employment of caustic issues had, he thinks, removed the diseased condition of the bones. The merit of the resting-plan was, therefore, limited to expedite the cure; for which intention he thinks it well adapted, where "softness prevails in the bones." In all the remainder, there was either no disease of the back, or it consisted of a muscular affection only. In confirmation of this assertion, he introduces

* Vol. xi. No. 41, *Edinburgh Medical and Surgical Journal.*
† Vol. x. No. 39.

accounts of three cases which fell under his own management. In these the spine was greatly bent, and the lower extremities more or less affected. One appeared to arise from hydrocephalus internus. The second occurred to a military officer.

"On the day after exposure to great fatigue and being wet through for many hours, he had an attack of fever, and, on attempting to walk, he found stiffness in his legs and inability to move them. Soon afterwards he was incapable of expelling his urine, and his feces at times passed involuntarily. That these symptoms gradually increased, until the lower half of his body was completely palsied." He had suffered four years when Mr. Earle was consulted. He was miserably helpless. His lower extremities were cold and benumbed, and he had lost all power of directing his steps. The whole vertebral column was slightly arched in the form of a half-hoop, but no part was unnaturally exuberant. When supported, he could raise his body from its bent shape to an upright position. The form of the back, when reclined, was quite natural. This circumstance, contrasted with subsequent experience, induces me strongly to believe that there was no disease whatever in the vertebral column; and this arching of the body was purely the effect of deficient muscular action, rendering the muscles of the back incapable of supporting the weight of the head and trunk: and, from the result of the examination which I was enabled to make a short time after, in a case which I shall presently relate, I am firmly of opinion that the deficient nervous energy in this case depended either on some morbid affection of the brain or its membranes, which rendered it incapable of transmitting its influence to the extreme parts of the body, or some diseased affection of the medulla spinalis, wholly independent on any disease in the vertebræ."

The third patient was a soldier, whose constitution had suffered from previous residence in hot climates. "In addition to the curved back, he had deficient muscular power in the lower limbs. There was a numbness and irregular action in the muscles of the fore-arms and hands: he was incapable of feeling any minute objects, and would often let things fall from his hands. On dissection, the liver, spleen, and lungs, were found to be diseased. "As I was most anxious to ascertain the state of the vertebræ and medulla spinalis, I did not pay much attention to these appearances, and, having cleared away the viscera, proceeded to make an accurate inspection of the vertebral column; and, no disease being apparent externally, I removed all the bodies of the vertebræ, by sawing through the rings between the articular processes. By this plan, I obtained a complete and very satisfactory view of the spinal marrow; but still no cause for the symptoms presented itself, and I was left to conjecture respecting the nature of the malady. On examining the head, however, the source of the mischief became apparent. The cellular structure of the pia mater was loaded with fluid, and the tunica arachnoidea was of a milky colour and thickened, especially at the base of the brain, where it was firmly connected with the pia mater, forming together a tough dense membrane. I am aware that to many this may appear an insufficient cause for the violence and extent of the symptoms; but, having paid considerable attention to the morbid anatomy of the brain, and well knowing the infinite variety of symptoms produced in different individuals by the same morbid alteration of structure, I am satisfied in my own mind that all the phenomena were referable to this diseased state of the membranes."

Whatever difference of opinion may arise in other respects, every one will, I think, admit Mr. Earle's cases to be legitimate examples of paraplegia, though certainly not of the variety described by Mr. Pott.

The more we become acquainted with spinal pathology, the stronger is our conviction that paraplegia ought to constitute a generic term, and include under it numerous species. Of these, one, and that comparatively of rare occurrence, originates in a carious state of the bones. In others, it takes place in the progress of the complaint; but in the greater number it never appears, though the health be generally impaired and destroyed by the mischievous effects impressed upon the constitution. We find the most essential signs of the disorder clearly displayed in Mr. Baynton's cases. There is debility, numbness, a pricking sensation, and torpor of the lower extremities, with incapability of directing the motions. In some, the upper limbs were also affected, as well as the internal viscera, giving to the malady a greater range of morbid action.

These phenomena having by all physiologists been referred to the nervous system, I do not think it necessary to produce any arguments in support of an admitted doctrine. The only question for discussion, is the seat of paraplegia. Is it ever in the brain, or is it always in the spinal marrow? In order to arrive at a more satisfactory conclusion, we may premise that none of the cerebral nerves, with the exception of the eighth pair, and perhaps the great sympathetic, descend below the throat; and the spinal marrow is united to the brain by means of the medulla oblongata. The cerebral nerves are principally employed to convey impressions to the sensorium commune, and transmit the determinations of the will to the muscles of voluntary motion. By means of the spinal nerves, the offices of secretion and excretion are performed. They regulate the circulation of the blood, and impart energy to the extremities of the body. These nerves are chiefly independent of the brain; for, if the head be cut off, in certain warm-blooded animals, and respiration artificially carried on, the trunk will live for many hours: but, destroy the spinal marrow, and the animal dies almost immediately. We are led from these experiments to conclude, that the nerves of the brain and spinal marrow discharge separate and distinct offices in the animal economy, though the brain certainly exercises its control, through the spinal cord, over the voluntary muscles. The involuntary motions are executed by the spinal cord alone, and are wholly independent of the brain.

The difference between the two orders of nerves is very apparent in the paralysis which they occasion. Hemiplegia is confined to one side; paraplegia equally affects both. In the former, the diseased muscles are more flabby, soft, and unresisting, than in the latter. The mental faculties are always disturbed in hemiplegia; in paraplegia they remain free. The latter complaint deranges some of the internal functions; but in the former they are little impaired. By paying attention to these several distinguishing characters, a clear diagnosis may generally be established between these different forms of palsy.*

If we examine Mr. Earle's first case according to the above rule, we shall find considerable derangement within the body, as well as in the lower extremities. I cannot therefore hesitate to consider it as a species of paraplegia, induced by compression of the spinal nerves. From the torpor of the bladder and rectum, together with the involuntary discharge of feces after taking purgative medicines, I am induced to place the seat of disease in the loins or sacrum. I have come to this conclusion because the nerves distributed to the rectum and bladder issue from these parts. Had the disorder occupied a higher station, some of the abdominal viscera would have participated in the complaint. I am of opinion that his fever after exposure to wet and fatigue, proceeded from an attack of inflammatory rheumatism in the loins or sacrum.

The joints, the tendinous sheaths, bursæ mucosæ, and in general all parts furnished with fibrous structure, are liable to its invasion. The tendons of muscles are often attacked, but the fleshy bellies seldom or never suffer from this complaint. We find, on dissection, a glairy fluid effused, with increased thickness and morbid enlargement of the surrounding structure.

Rheumatism seizes indiscriminately upon the vertebral joints. The back is frequently the seat, because it is supplied with

* The diagnostic signs will be more fully considered in a future essay.

strong ligaments to provide for the weight and motions of the body. It has likewise the usual articular provisions, and a tendinous expansion, the common posterior ligament of the vertebræ, enclosed within the theca spinalis. Rheumatic inflammation of these organs, by enlarging their bulk and dimensions, occasions compression upon the spinal cord, impedes its operations, and thus induces coldness, debility, numbness, loss of feeling and motion in the limbs, and the various sensations and inabilities complained of in the internal parts. In agricultural districts, rheumatism is of frequent occurrence among the labouring poor, from their being thinly clothed and so much exposed to the changes of the weather. When it invades the back, symptomatic paraplegia often supervenes in the arms or legs. Many patients of this description have been admitted into the Horncastle Dispensary, under my professional care. In some, the paraplegia was entirely removed with the primary complaint: in others, it continued to torment the sufferer during the rest of life.

It is for these several reasons, and from the suddenness of the attack, that I feel inclined to impute the origin of Mr. Earle's first case to lumbago, rather than any other cause. Had the inflammation invaded bone or cartilage, I think its effects would not have appeared so early as the following day: I therefore coincide in the opinion that the vertebræ and cartilages were wholly untouched. The disorder in process of time became chronic, and was then attended with muscular debility. Still I think it began in membranous parts, and extended from them to the muscles. That the articulating organs of the vertebræ were relaxed and preternaturally elongated when Mr. Earle was consulted, must, I think, be conceded, from the arch or "half-hooped form" of the back in an erect posture, and its becoming straight upon lying down. To me it appears altogether impossible to distort the bones of the vertebral column in the manner described, without stretching and hurting some of the articulating connections. So long as they remain firm and unyielding, the vertebræ continue fixed; but, when they begin to lengthen, the vertebræ as certainly show a disposition to move out of the line: I therefore think it much more probable that the affection was situated in the ligaments than the muscles, especially since fibrous texture possesses little inherent contractibility. When once over-stretched, it recovers its lost tone slowly and with difficulty. In this respect it differs from muscles, which admit of considerable contraction and relaxation, without injury to their functions. The bent figure of the vertebral column, and consequent extension of the spinal marrow, would account for the symptoms under which the patient laboured when Mr. Earle saw him; but the commencement of disease on the morning after long exposure to wet and fatigue, cannot be referred to a circumstance which had not then taken place. The subsequent appearance of the same complaint in other vertebræ, is agreeable to an established law of the animal economy. Rheumatic inflammation passes successively from joint to joint, sometimes leaving the former to invade the latter; more frequently, it attacks the new organ without deserting the old one. It is no uncommon thing for sciatica to proceed to the knees, ankles, and superior extremities. After remaining in all of them for a certain period, it gradually disappears, or degenerates into a protracted rheumatism. It is in this way that we account for the membranes and ligaments of the back becoming so generally and obstinately disordered in Mr. Earle's and Mr. Baynton's different cases.

An objection may be raised against this explanation, because, in the second case, Mr. Earle found no disease of the spinal marrow, after viewing it by "sawing through the rings between the articular processes." It is clear from the statement, that it never occurred to his mind to examine the articulations with any particular attention; otherwise, he would have laid them open with greater care and in a different manner. His eyes and thoughts were exclusively directed to the vertebral bones, their intermediate cartilages, and the spinal cord; and, to get to the objects of research, he incautiously tore and mangled the articulating membranes with a coarse instrument. It could not be expected, after such treatment, that the parts would display any strong signs of diseased structure; and, therefore, his finding no sufficient cause for the symptoms in the spinal marrow, does not convince me that none were present in the contiguous ligaments. The advocates for pressure generally would have examined the bones, cartilages, and membranes which surround the spinal marrow or connect the parts together, under an idea that paraplegia derives its origin from disease situate sometimes in one tissue and sometimes in another.

I have thus endeavoured, in controverting the theory of Mr. Earle, to establish the phenomena on a basis ample enough to afford them support, and more conformable to the principles of the animal economy.

Dr. Baillie has also favoured us with his sentiments on paraplegia, in the Transactions of the Royal College of Physicians.* It is much to be regretted, however, that he did not superintend the dissection of a patient, whom he had occasionally visited during life, and whose case constitutes the basis of his paper: had he personally directed the investigation, I am convinced that the spinal marrow and its membranes would not,

* Vol. vi. page 16.

under such circumstances, have escaped the notice of a physician intimately acquainted with morbid anatomy and the general principles of his profession. "This last form of paralytic affection," (the paraplegia), he observes, "has, as far as I can judge from individual experience, increased considerably in this country within the last fifteen or twenty years, although it be very difficult to assign any satisfactory reason for it. Paraplegia in adults has been considered by most medical men as being produced by some disease, either in the bones or ligaments of the spine, or in the cavity of the spine, most commonly at the loins, independently of any disease in the brain. The reason of this general opinion has probably been, that the lower part of the body from the loins downwards is, in this disease, affected with paralysis; and that, in children, a similar disease is often obviously dependent upon a morbid affection of the lumbar or some other portion of the spine. In adults, however, where there has been no accident affecting the spine by outward violence, paraplegia, I believe, depends most commonly, in a great measure, upon a disease affecting the brain itself. This opinion I have entertained for several years, and some other medical men have likewise held the same opinion. It is, however, by no means general; and the chief object which I have in view in writing this paper, is to render this opinion more commonly known, that it may either be established or be properly limited by the future observations of other practitioners."

The brain and its membranes, in the case recorded, exhibited strong marks of disease, though they were not, as I think, sufficiently distinguishing to account for the paraplegic symptoms. We are not informed how the mental faculties and organs of sense were exercised under the pressure of the disease, nor to what degree the feeling and motion of the arms were impaired, or in what manner the thoracic and abdominal viscera discharged their respective offices. These omissions are the more to be lamented, because, if the several functions remained undisturbed, we have little reason for imputing the disorder of the lower limbs to the cause assigned.

"A considerable quantity of water," we are told, "was discharged during the inspection from within the theca of the spinal marrow." When such an obvious source of the disease presented itself, we have surely more reason to refer the paraplegia to it, than attempt to search for its origin in the deranged structure of a distant part. We have the authority* of celebrated names for maintaining that, when a fluid is found after

* Morgag. Epist. xii. art. 9; Willis; Lieutaud, lib. iv. p. 341; Bonetus Magnetus; Coiterus.

death both in the head and spinal tube, it sometimes commences in one part and sometimes in the other. Since, then, the effused liquid may, in the case recited, have had a two-fold commencement, it is impossible to determine, imperfectly as the inspection was conducted, where it was really formed. Had the surgeons examined the vertebral column with proper care, I think they would have discovered in it the origin of the disease, and that the malady in the skull either proceeded from the spine, or was only a co-existent and unconnected attendant. We feel more grieved that the state and condition of this organ and its contents should have been overlooked, because the subject has been very little elucidated by careful dissection, and appears to be much in want of further investigation.

We have thus given a brief analysis of the works of Mr. Pott and Mr. Baynton, the two chief practical authorities on this subject, interspersing our remarks with the observations of other writers. This was undertaken with a view to communicate what has hitherto been advised and done in respect to one of the most obstinate and distressing calamities to which the human frame is exposed,—a calamity which poisons our present happiness, and deprives us of the hope of future enjoyment.

